# The effect of food on the pharmacokinetics of oral fluralaner in dogs

**DOI:** 10.1186/1756-3305-7-84

**Published:** 2014-03-05

**Authors:** Feli M Walther, Mark J Allan, Rainer KA Roepke, Martin C Nuernberger

**Affiliations:** 1 MSD Animal Health Innovation GmbH, Zur Propstei, 55270 Schwabenheim, Germany

**Keywords:** Fluralaner, Dog, Pharmacokinetics, Food effect, Fasted

## Abstract

**Background:**

Fluralaner is a novel systemic ectoparasiticide for dogs providing long-acting flea- and tick-control after a single oral dose. The pharmacokinetics of orally administered drugs may be influenced by feeding. This study investigated the influence of concurrent feeding on fluralaner pharmacokinetics.

**Methods:**

Twelve fasted or fed beagles received a single oral administration of 25 mg fluralaner/kg body weight in a chewable tablet. Plasma samples were collected at multiple post-treatment time points for fluralaner concentration analysis. Clinical observations were performed on all dogs at regular intervals throughout the study.

**Results:**

Fluralaner was readily absorbed in fasted and fed dogs administered at a dose of 25 mg/kg BW with a similar mean t_max_ for both groups. In fed dogs, AUC and C_max_ were increased compared to fasted dogs by a factor of 2.5 and 2.1 respectively. The difference in AUC and C_max_ between the fed and fasted groups was statistically significant. No adverse events were observed following oral fluralaner administration to fasted and fed dogs.

**Conclusions:**

Fluralaner is absorbed to a considerable extent in fasted and fed dogs. Administration of fluralaner chewable tablets with food significantly increases bioavailability.

## Background

Fluralaner is a novel systemically administered insecticidal and acaricidal compound that provides long-acting efficacy after oral administration to dogs. Fluralaner belongs to a new class of compounds, the isoxazolines. A field study has shown that a single fluralaner dose administered orally to dogs provides at least twelve weeks of flea- and tick-control [1]. The long duration of activity offers a more convenient treatment over monthly flea and tick control treatments with a potential compliance advantage, reducing the risk of vector-transmitted diseases.

Feeding affects gastrointestinal physiology and therefore may influence the pharmacokinetics of a concurrently administered drug through reduced, delayed, increased, or accelerated absorption; in addition, there are drugs with no food interaction [2-4]. Altered pharmacokinetics may have an impact on the clinical activity of fluralaner [3,4]. For example, an increase in absorption in a fed animal may lead to a prolonged period of efficacy. Administration of fluralaner tablets at or around the time of feeding may be an option chosen by some dog owners to facilitate administration of the chewable tablet. A drug-food effect cannot be predicted on a scientific basis and specific studies are required to investigate possible effects [2-4]; therefore, this study was performed to evaluate the impact of food on the pharmacokinetic profile of fluralaner chewable tablets.

## Methods

This study was conducted in compliance with the German animal protection legislation framework and ethical approval was obtained before the start of the study (authorization reference 23 177-07 /G 08-4-003). Dogs were kept indoors in pens with sealed floors and individually housed until 3 days after tablet administration. They had access to water *ad libitum* throughout the study period and were fed a standard dog diet (Bosch Tiernahrung GmbH&Co. KG; dry kibble food; composition: protein 21%, fat 6%, crude fiber 7%, crude ash 6%, moisture 10%). 12 healthy beagles were allocated to either a *fed* or *fasted* study group by sorting dogs within gender according to ascending body weight and alternately assigning dogs to a group (Table 1).

**Table 1:** Fasted and fed dog groups for evaluation of fluralaner pharmacokinetic parameters

		**Fasted group**	**Fed group**
Gender	Male	4	3
	Female	2	3
Body weight (kg)	Mean	14.0	13.5
	Range	12.2 – 16.6	12.0 – 15.6

All dogs were dosed once orally with 25 mg fluralaner/kg body weight on day 0 using chewable tablets containing fluralaner. Chewable tablets were cut to achieve the individual target dose. Tablets were placed on the back of the tongue and swallowing was stimulated with a small amount of tap water.

Both groups were fasted for 25 hours prior to fluralaner administration. Dogs in the *fed* group were offered half the normal daily food ration 15 minutes prior to fluralaner administration and the remaining half of the daily food ration was offered to these dogs immediately after administration. Dogs in the *fasted* group received orally administered fluralaner chewable tablets without feeding and remained unfed for a further 8 hours. Dogs were observed at regular intervals for any clinical findings throughout the study. The veterinary study supervisor assessed any clinical findings for their relationship to fluralaner treatment. All treatment-related findings were classified as adverse events.

Blood samples for fluralaner plasma concentration determination were collected prior to tablet administration and at 2, 4, and 8 hours, and then 1, 2, 3, 4, 7, 14, 21, 28, 42, 56, 70, 84, and 91 days after tablet administration. The blood sampling time points were selected based on previous pharmacokinetic data (unpublished observations), to cover initial rapid absorption, redistribution and prolonged elimination over 13 weeks following oral treatment. Fluralaner blood plasma concentrations were determined using automated solid-phase extraction coupled to liquid chromatography with mass-spectrometry (Online-SPE – HPLC/MS/MS; lower limit of quantification 5 ng/ml). The bioanalytical method was validated based on regulatory guidelines [5,6].

Fluralaner pharmacokinetics evaluation was based on the plasma concentration of the parent compound for the area under the curve from time 0 to the last sampling time point associated with a quantifiable concentration (91 days after administration, AUC_0-91d_), maximum plasma concentration (C_max_) and time to C_max_ (t_max_). Pharmacokinetic parameters were calculated using noncompartmental methods (WinNonlin 5.2.1, Pharsight, Mountain View, California). The AUC_0-91d_ was calculated using the linear trapezoidal method.

The effect of food on fluralaner pharmacokinetics was calculated by comparison of the means of AUC_0-91d_ and C_max_ of both groups according to the following ratios: AUC_0-91d(fed)_/AUC_0-91d(fasted)_ or C_max(fed)_/C_max(fasted)_. Statistics (SAS® Language: Reference, Version 9.3, SAS Institute Inc., Cary, NC, USA) were calculated using the individual dog as the experimental unit.

## Results and discussion

**Figure 1:**
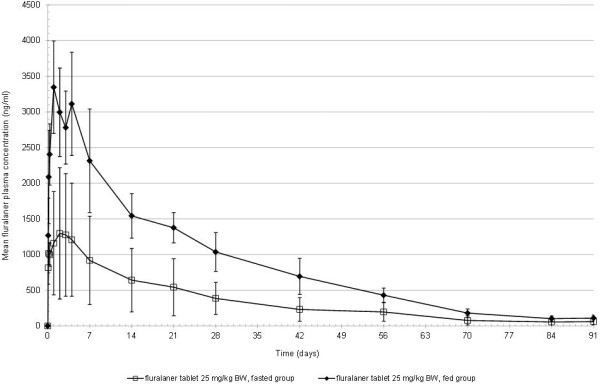
Mean fluralaner plasma concentration (± standard deviation) following oral administration (25 mg/kg) to fasted or fed dogs.

Orally administered fluralaner was readily absorbed (mean T_max_ of 1 day) in both fasted and fed dogs and was quantifiable in both groups throughout the entire study period (Figure 1). The calculated AUC_0-91d_ ratio between the two groups was 2.5 and the C_max_ ratio was 2.1; AUC_0-91d_ and C_max_ were significantly higher in the fed group (Table 2). In contrast, t_max_ was not significantly different between both study groups (Table 2). Many drugs that show food interactions as increased absorption also exhibit delayed absorption [2]. In the present study, the similarity between fed and fasted groups in the time to reach maximum plasma concentrations indicates that fluralaner absorption, and thereby onset of the parasiticide effect, is not delayed through feeding.

**Table 2:** Fluralaner pharmacokinetic parameters following oral administration (25 mg/kg) to fasted or fed dogs

**Parameter**	**Unit**	**Fasted group**	**Fed group**	**P value**	**Ratio (fed/fasted)**
		Mean ± SD	Mean ± SD		
		(range)	(range)		
C_max_	(ng/mL)	1591 ± 708	3377 ± 669	0.0015	2.1
		(1037–2683)	(2325 – 4326)		
t_max_	(day)	1.3 ± 1.2	1.4 ± 1.3	0.8096	-
		(0.17 – 3.00)	(0.33 – 4.00)		
AUC_0-91d_	(day*ng/mL)	31369 ± 20654	78785 ± 11440	0.0022	2.5
		(14316 – 63557)	(60862 – 91854)		

SD: Standard deviation.

Food can also effect drug distribution, metabolism and elimination [3,4]. However, the similar pattern of plasma concentration in both groups of the present study (Figure 1) indicates that the feeding status of the dog has no effect on subsequent distribution, metabolism or excretion of fluralaner.

The extent of a drug-food interaction can be affected by the drug formulation [2,3]. However, the tablet formulation used in the present study is equivalent to the formulation developed for the final commercial product Bravecto^TM^; therefore, the results of the present study are considered to represent the drug-food interaction for Bravecto^TM^.

For some drugs, bioavailability is increased with an increase in dietary fat content, whereas dietary fiber may reduce drug availability [3]. For this study, a commercially available low-fat diet with high fiber content was used with the expectation that this would potentially minimize the effect of the diet on fluralaner availability. However, the food effect on fluralaner may be similar for other types of diets.

The timing of feeding relative to oral treatment may impact drug-food interactions [3]. In the present study, all fed group dogs completely consumed the initial portion of daily food ration prior to fluralaner administration and rapidly consumed the remaining daily ration offered after administration. Therefore, the recommended administration for Bravecto^TM^ chewable tablets is at or around the time of feeding.

Co-administration of a drug with food might reduce or increase inter-individual variability in drug plasma concentrations [3]. In the present study, both, the AUC and C_max_, showed lower standard deviations in the fed group, indicating less pronounced individual variability and providing an additional reason to recommend co-administration with food.

This study did not evaluate the impact on fluralaner pharmacokinetics of feeding quantities smaller than the full daily ration at the time of treatment; however, it is possible that co-administration with smaller quantities of food will also increase fluralaner bioavailability.

In the present study, no clinical findings were observed in dogs in either group post treatment indicating an adverse event; therefore, the increase in fluralaner bioavailability is not thought to be associated with an increased risk of toxicity, as has been suggested for other compounds [4]. In support of this, fluralaner safety following high dose oral administration was thoroughly investigated, confirming a high safety margin for fluralaner [7].

## Conclusions

Fluralaner is a novel potent insecticide and acaricide with a longer duration of action in dogs than currently available commercial products applied orally or topically [1]. Fluralaner is formulated as an oral chewable tablet and some dog owners may choose to facilitate administration by dosing at or around the time of feeding. The results of this study show that fluralaner is rapidly absorbed in both fasted and fed dogs and is detectable in plasma for more than 12 weeks in both groups. Oral administration of fluralaner to dogs together with their full ration of food resulted in total drug exposure over the subsequent 91 days that was 2.5 times greater than in dogs treated while fasting. Similarly, the maximum plasma concentration was 2.1 times higher in fully fed dogs compared with fasted dogs.

Administration at the time of feeding more than doubles fluralaner bioavailability and this difference is statistically significant. For these reasons, administration of fluralaner chewable tablets at or around the time of feeding is recommended.

## Competing interests

FMW, MJA, RKAR and MCN are employees of Merck/MSD Animal Health.

## Authors’ contributions

FMW, MJA, RKAR and MCN authored the study design, monitored the study and interpreted the results. All authors revised and approved the final version of the manuscript.
